# Neoantigenic properties of *TP53* variants influence cancer risk in individuals with Li-Fraumeni syndrome

**DOI:** 10.1016/j.ebiom.2025.106065

**Published:** 2025-12-09

**Authors:** Emilie Montellier, Olivier Manches, Jonathan Gaucher, Claire Freycon, David Hoyos, Sandrine Blanchet, Murielle Verboom, Christina M. Dutzmann, Sophie Coutant, Jacqueline Bou, Bertrand Fin, Robert Olaso, Jean-François Deleuze, Thierry Frébourg, Benjamin D. Greenbaum, Arnold J. Levine, Christian P. Kratz, Gaëlle Bougeard, Pierre Hainaut

**Affiliations:** a Université Grenoble Alpes, Inserm 1209, CNRS 5309, Institute for Advanced Biosciences, F38000, Grenoble, France; b EFS, R&D Department, Grenoble, France; c Grenoble Alpes University, HP2 Laboratory, INSERM U1300, Grenoble Alpes University Hospital, Grenoble, France; d Department of Pediatric Hematology-Oncology, Grenoble Alpes University Hospital, Grenoble, France; e Halvorsen Center for Computational Oncology, Department of Epidemiology and Biostatistics, Memorial Sloan Kettering Cancer Center, New York, NY, USA; f Institute of Transfusion Medicine and Transplant Engineering, Hannover Medical School, Hannover, Germany; g Pediatric Hematology and Oncology, Hannover Medical School, Hannover, Germany; h Univ Rouen Normandie, Inserm U1245, Normandie Univ, CHU Rouen, Department of Genetics, F-76000, Rouen, France; i Université Paris-Saclay, CEA, Centre National de Recherche en Génomique Humaine (CNRGH), 91057, Evry, France; j Simons Center for Systems Biology, Institute for Advanced Study, Princeton, NJ, USA; k Physiology, Biophysics & Systems Biology, Weill Cornell Medicine, Weill Cornell Medical College, New York, NY, USA; l The Olayan Center for Cancer Vaccines, Memorial Sloan Kettering Cancer Center, New York, NY, USA

**Keywords:** *TP53* variants, Li-Fraumeni syndrome, Genetic cancer predisposition, Neoantigenicity, Genotype-phenotype correlation

## Abstract

**Background:**

Li-Fraumeni Syndrome (LFS) is a heterogenous cancer predisposition condition caused by pathogenic *TP53* variants, characterised by a lifelong high risk of a broad spectrum of cancers. Certain pathogenic *TP53* variants have been shown be immunogenic in a somatic context. Whether neoantigenicity contributes to LFS heterogeneity is unknown. In this study we analysed the correlations between predicted neoantigenic properties of pathogenic *TP53* missense variants and LFS phenotypes.

**Methods:**

MHC-I presentation scores were generated for the set of nonameric neo-peptides surrounding each *TP53* missense variant against 145 different HLA-I using NetMHCpan 4.1 and the Allele Frequency Net Database. A predicted neoantigenic score (PNS) was calculated for each variant. Association study was performed between PNS, LFS presentation and individual HLA-I genotyping, in individuals carrying *TP53* germline pathogenic variants using data from mutation databases and clinical registries. Genotype-phenotype data were leveraged from the public *TP53* database (germline dataset, n = 3446; https://tp53.isb-cgc.org/) and two independent LFS clinical registries (n = 339). Individual correlations between HLA-I genotyping, *TP53* missense variants and phenotypes were investigated in a group of 173 subjects with LFS.

**Findings:**

Among individuals with frequent *TP53* pathogenic variants, PNS was strongly correlated with median age at first cancer (range 18–43 years, R = 0.69, p = 0.0132). Compared to individuals with low PNS (<1) variants, those with high PNS (>2) variants showed delayed median age at first diagnosis (34 years vs. 25 years, p = 0.0009), fewer sarcomas (osteosarcoma [RR 0.29, p = 0.02]; soft-tissue [RR 0.41, p = 0.02]), and more cancer types typically not associated with LFS spectrum [RR 1.61, p = 0.02].

**Interpretation:**

MHC-I neoantigenic properties of *TP53* variants are associated with differences in cancer risk and spectrum in individuals with pathogenic *TP53* variants, suggesting that individual variant-specific immune response could contribute to the heterogenous presentation of LFS.

**Funding:**

10.13039/501100000780European Commission, Fondation MSDAvenir, 10.13039/501100002347BMBF, 10.13039/501100007311Deutsche Kinderkrebsstiftung, 10.13039/501100008530European Regional Development Fund, 10.13039/501100018696Région Normandie, 10.13039/100000002NIH, 10.13039/100014599Mark Foundation, and 10.13039/100005883Hertz Foundation.

**Research in context**

**Evidence before this study**

Li-Fraumeni syndrome (LFS) is a rare and highly penetrant cancer predisposition syndrome caused by pathogenic *TP53* germline variants. Previous studies have shown considerable clinical heterogeneity among patients, including variation in cancer types and age at onset, which is not fully explained by *TP53* variant type alone. Somatic studies have revealed that some *TP53* missense variants generate immunogenic neoantigens presented by MHC-I, but the potential role of neoantigenicity in inherited cancer syndromes such as LFS has not been investigated.

**Added value of this study**

This study shows that predicted MHC-I neoantigenic properties of *TP53* missense variants are associated with age of cancer onset and tumour spectrum in individuals with LFS. By integrating *in silico* neoantigen predictions with population-based HLA-I allele frequencies, curated databases, and individual-level genotyping data from clinical registries, we identify a strong correlation between neoantigenic potential and clinical phenotype. Our results indicate that immunogenic variants are associated with later cancer onset and a shift in tumour spectrum toward non-LFS-associated cancers, consistent with a potential role of immune-mediated tumour suppression.

**Implications of all the available evidence**

Our findings highlight immunogenicity as a factor associated with variation in cancer risk and presentation in LFS. These results provide a rational for further investigation of neoantigen-based risk stratification in hereditary cancer syndromes and could ultimately inform personalised cancer surveillance strategies integrating both germline variant properties and HLA background. This work also suggests a broader conceptual framework for understanding genotype–phenotype variability in other germline cancer predisposition genes.

## Introduction

Li-Fraumeni syndrome (LFS; MIM #151623) is a severe cancer predisposition condition linked to pathogenic germline or postzygotic mosaic *TP53* variants, 80% of which are missense.^1^ LFS typically unfolds in three phases, 1- childhood (0–17 years), marked by adrenocortical carcinoma (ACC); choroid plexus tumour (CPT); medulloblastoma (MB), and rhabdomyosarcoma (RMS) in infancy, other soft tissue sarcoma (STS), osteosarcoma (OS) and central nervous system (CNS) gliomas in adolescence; 2- early adulthood (18–45 years), dominated by premenopausal breast cancer (PBC), STS and glioma; and 3- a late adulthood (over 45 years), with increased risk of adenocarcinoma (LUAD), STS (typically leiomyosarcoma), colorectal cancer and prostate cancer.^2^ This broad spectrum is captured in the revised Chompret criteria^1^^,^^3^^,^^4^ used for identifying patients and relatives for *TP53* mutation testing.

The p53 protein is a transcription factor orchestrating complex cellular and systemic anti-proliferative responses.^5^, ^6^, ^7^ Missense mutations cause structural change that disrupts DNA binding and impair transcriptional activity. Moreover, many p53 proteins become intrinsically stable and accumulate, causing dominant-negative or gain of function effects.^8^

Recent work by Hoyos et al.^9^ revealed that the neoepitopes formed by somatic *TP53* variants could induce CD8+ T cells responses, suggesting mutant p53 “fitness” was the result of a trade-off between oncogenicity and immunogenicity, with preferential selection of variants predicted to be poorly immunogenic. This correlation supports that variant immunoediting contributes to the selective pressures that shape cancer development and progression.

In this study we examined whether predicted neoantigenic properties of germline *TP53* missense variants modulate LFS cancer phenotypes. Using NetMHCpan 4.1 and the Allele Frequency Net Database,^10^^,^^11^ we estimated neoantigenic prediction scores for 2314 *TP53* missense variants (Kato dataset, representing all possible amino acid substitutions caused by a single point mutation) across 145 HLA-I alleles, encompassing all missense variants reported to date in subjects and families matching LFS criteria. We next analysed correlations with cancer phenotypes (age at diagnosis, cancer types) in a large public dataset of individuals carrying germline *TP53* variants^12^ and in a validation cohort based on two clinical LFS registries (n = 339).^1^^,^^13^ Finally, we examined the individual correlations between HLA-I alleles, variants and phenotypes in a subset of 173 patients from the validation cohort. We show that variants predicted to have high neoantigenic scores were associated with delayed cancer onset and attenuated LFS phenotypes, suggesting that variant immunoediting influences individual LFS presentation.

## Methods

Figure S1 provides a flowchart summarizing the study design.

### Predictions of *TP53* variant neoantigenicity

We developed a predicted neoantigenicity score for 2314 missense *TP53* variants previously classified into 4 classes according to their transactivation properties in a yeast-based functional assay (from class A, most severe functional disruption, to class D, less severe functional disruption based on transactivation properties).^14^ For each variant, nine 9-mer peptides were assessed via NetMHCpan 4.1 (https://services.healthtech.dtu.dk/services/NetMHCpan-4.1/) for binding affinity to 145 HLA-I alleles.^10^ Metrics included: 1- Minimal Affinity Score (MAS), the best predicted 9-mer peptide/HLA-I pair (in nanomol, nM), Affinity ≥500 nM was excluded as non-relevant,^10^ 2- HLA Count Score (HCS), the number of responsive HLA-I alleles, 3- World Coverage Score (WCS), the estimated population-wide HLA-I coverage from the Allele Frequency Net Database (http://www.allelefrequencies.net/),^11^ and 4- Amplitude Score (AMS), the ratio of mutant-to-wild-type peptide affinity for a given HLA-I.^15^ The three metrics MAS, HCS and WCS showed strong pairwise correlation (Spearman's rho ≈ ±1, p < 0.0001 for all comparisons) and were combined into a Predicted Neoantigenic Score (PNS), calculated as the sum of the three normalised [0–1] metrics (with MAS inverted prior to normalisation). PNS was aggregated in 3 categories (low, PNS <1; intermediate, PNS 1–2; high, PNS >2). Low PNS values correspond to reduced predicted neoantigen recognition of the variant peptides, whereas high PNS values indicate increased predicted neoantigen recognition. Table S1 presents the predicted neoantigenicity scores for the 2314 *TP53* variants, while Table S2 lists the 145 HLA-I alleles included in the analysis.

### Correlations between *TP53* genotypes, neoantigenic prediction scores and LFS phenotypes

LFS phenotypes were leveraged from the NCI/IARC *TP53* germline database R20^12^ (https://tp53.isb-cgc.org) after classifying variants into functional classes A to D.^14^ Overall, 144 class A variants were retrieved in 1426 individuals. The validation cohort consisted of 89 class A variants found in 339 patients from French and German LFS registries.^1^^,^^13^ The NCI/IARC database and validation cohorts overlap minimally, owing to previously published cases.

### Individual correlations between *TP53* variants, HLA-I and LFS phenotype

HLA-I genotypes were inferred from Whole Exome Sequencing data in 173 patients from the validation cohort (Table S3). Patients were stratified in 4 groups based on MAS: (1) ≤10 nM, high affinity HLA-I binding; (2) >10 nM and <250 nM, intermediate; (3) over >250 nM, low; and (4) no relevant HLA-I binding.

### Statistics

Graphics and statistical analyses were conducted in Rstudio (version 2022.12.0). Normality of continuous variables was assessed using the Shapiro–Wilk test. Pearson correlation was used to evaluate linear relationships when both variables were normally distributed, whereas Spearman correlation was applied when at least one variable was not normally distributed or when the relationship was potentially non-linear and monotonic. Global Log-rank tests were first performed to assess overall differences between survival curves. Pairwise Log-rank tests with Bonferroni correction were then employed to compare survival curves across groups while controlling for multiple comparisons. Pairwise Chi-square tests with Benjamini-Hochberg (FDR) correction were used to evaluate differences in tumour type frequencies across groups, controlling for the expected proportion of false positive findings while maintaining adequate statistical power. To further quantify the strength of association between groups and tumour distribution, logistic regression models were applied to estimate risk ratios (RR) with 95% confidence intervals (CI). For pairwise comparisons of median age of different tumour distributions across groups, Wilcoxon rank sum tests with Benjamini-Hochberg (FDR) correction were applied, controlling for the expected proportion of false positive findings while maintaining adequate statistical power. For all statistical tests, 0.05 was used as the threshold of p-value (p) significance. This study was observational and based on existing clinical and genomic datasets. All eligible cases meeting inclusion criteria (individuals carrying germline pathogenic *TP53* variants with annotated clinical data) were included. Sample sizes were determined by data availability rather than by a priori statistical power calculations.

### Ethics

Written informed consent was obtained from all patients (or parents/legal guardians) included in the validation cohort, in compliance with legislation in France (CCTIRS (Comité Consultatif sur le Traitement de l'Information en matière de Recherche dans le domaine de la Santé; N°07.291) and CNIL (Commission Nationale de l'Informatique et des Libertés; N°907262) or Germany (institutional review board–approved German Cancer Predisposition Syndrome Registry; DRKS00017382).

### Role of funders

The funding sources had no role in the study design, data collection, data analysis, data interpretation, writing of the report, or in the decision to submit the paper for publication. The corresponding author had full access to all the data in the study and had final responsibility for the decision to submit for publication.

## Results

### Mapping of predicted neoantigenic scores across *TP53*

**Fig. 1:**
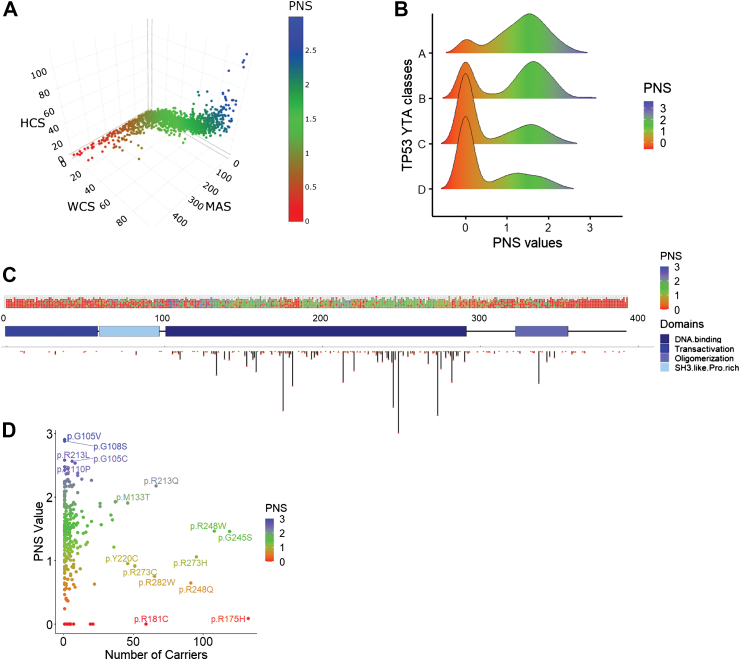
**Mapping of the Predicted Neoantigenic Score (PNS) for *TP53*. A.** Mapping of the Minimal Affinity Score (MAS), the World Coverage Score (WCS), and the HLA Count Score (HCS) for each of the 2314 *TP53* missense variants. Spearman's rho ≈ ±1, p < 0.0001 for all comparisons. The 3 scores were normalised from 0 to 1 and an integrated score based on the sum of the 3 normalised scores was built (Predicted Neoantigenic Score, PNS, ranging from 0 to 3), and is represented with the colour scale. **B.** Repartition of the PNS within the *TP53* variants classes (A, B, C and D). The density of PNS is displayed for each *TP53* variants class (YTA classes, from Montellier et al.^14^). Variants A, n = 402; B, n = 208; C, n = 671; D, n = 1033). Correlation of PNS with variant classes (Chi-square test, p < 0.001). **C.** Repartition of the PNS along p53 protein structure. Upper panel represents the PNS values at each p53 amino acid. The middle panel represents p53 secondary structure with codon numbers. The lower panel represents the count of individuals carrying germline *TP53* variants at each amino acid (NCI/IARC germline database). **D.** Relationship between the number of individuals with germline *TP53* variants and the PNS value for each *TP53* variant.

Of the 2314 variants analysed, 1185 had a peptide binding at least one HLA-I with affinity <500 nM (Table S1). Fig. 1A and B shows the correlations between the three components of PNS (Spearman's rho ≈ ±1, p < 0.0001) and its distribution across each of the 4 variant functional classes, highlighting a correlation with variant severity from D (more benign variants, lower PNS) to A (more severe variants, higher PNS) (Chi-square test, p < 0.001), consistent with severe variants causing more disruptive and neoantigenic substitutions. Variants with Intermediate (I) or High (H) PNS tended to cluster within the p53 protein DNA-binding and oligomerization domains (Fig. 1C). Common (hotspots) LFS variants had low (L) PNS (Fig. 1D) whereas the 10 variants with the highest PNS (≥2.5) were rare or absent in the NCI/IARC *TP53* database (9/3446 individuals, 0.26%). These observations suggest negative selection against strongly immunogenic mutations.

### Variable cancer risk patterns among frequent *TP53* variants correlate with neoantigenic potential

**Fig. 2:**
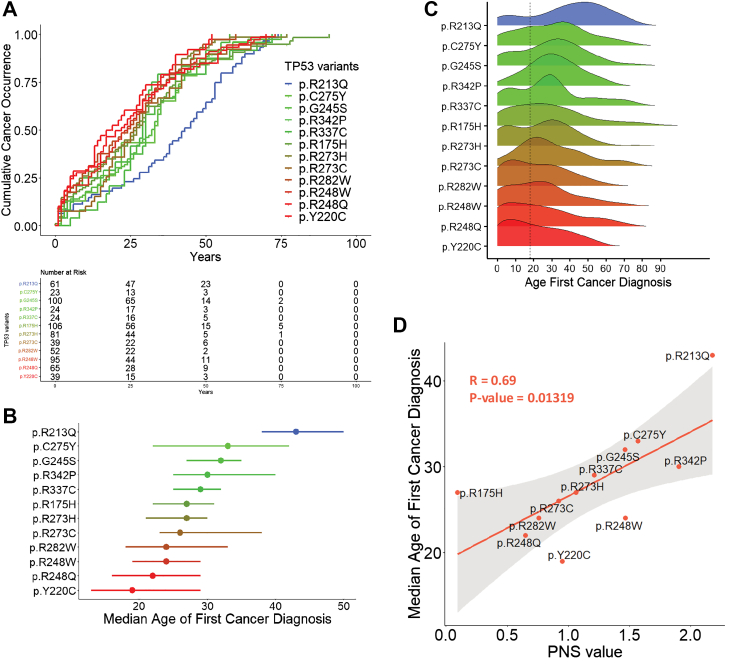
***TP53* germline variants display variable cumulative cancer occurrence patterns, which are correlated with the PNS. A.** Variable cumulative cancer occurrence patterns of frequent *TP53* variants from class A. Variants present in at least 20 individuals were considered to conduct this analysis. Number of individuals: p.Y220C, n = 39; p.R248Q, n = 65; p.248 W, n = 95; p.R282W, n = 52; p.R273C, n = 39; p.R273H, n = 81; p.R175H, n = 106; p.R337C, n = 24; p.R342P, n = 24; p.G245S, n = 100; p.C275Y, n = 23; p.R213Q, n = 61. The inverted Kaplan–Meier curves represent the age of diagnosis of the first cancer (n = 709 individuals with age information, top panel). A global Log-rank test was performed to assess differences between the curves (p = 0.0002). Number at risk table is displayed below the graph. **B.** Median age of first cancer diagnosis (dots) along with confidence interval at 95% (bars asides the dots) for each *TP53* variant. **C.** Pattern of age at first cancer diagnosis along lifetime. Density plot representing the pattern for each *TP53* variant. A vertical dotted line indicated age 18. **D.** Positive correlation of median age of first cancer diagnoses and the PNS value. For each frequent *TP53* variant, the median age of first cancer was plotted against the PNS value of the variant. The linear regression line is displayed in orange, and a Pearson correlation test was performed to assess relationship between the 2 variables (R = 0.69, p = 0.0132).

Class A consists of severe variants (n = 402) detected in 41% (1426/3446) subjects in the NCI/IARC *TP53* database, including 865 individuals carrying 12 “hotspot” variants.^14^ Despite their quasi-identical functional profiles, these variants are associated with significant differences in cancer accrual over lifetime (global Log-rank test, p = 0.0002) and in the median age at cancer diagnosis (from 19 years, (95% CI [13–29]) for p.Y220C to 33 years (95% CI [22–42]) for p.C275Y, with p.R213Q as an outlier at median age 43 years (95% CI [38–50])) (Fig. 2A–C). Among these variants, 5 had L PNS (p.R175H, p.R248Q, p.R282W, p.R273C, p.Y220C), 6 had I PNS (p.R273H, p.R337C, p.R248W, p.G245S, p.C275Y, p.R342P) and only 1 had H PNS (p.R213Q), outlining a strong positive correlation between median age at first cancer diagnosis and PNS (Pearson's R = 0.69, p = 0.0132) (Fig. 2D). Lifetime cancer accrual showed a progressive shift in relation with PNS, from early-life cancers (predominant for individuals with L PNS variants such as p.Y220C, p.R248Q, p.R282W), to adult-life cancers (predominant for individuals with I/H PNS such as p.G245S, p.C275Y and p.R213Q) (Fig. 2C).

Of note, whereas MAS, HCS, WCS were each significantly correlated with age at cancer diagnosis (|Spearman's rho| > 0.60, p < 0.05), AMS was not (Spearman's rho = 0.25, p = 0.43), suggesting that the latter metrics is not a significant predictor of *TP53* variant immunogenicity (Figure S2A). The immune fitness score developed by Hoyos et al.^9^ (FIT) and PNS showed a moderate negative correlation (Spearman's rho = −0.50, p < 0.0001), indicating that the two metrics are related (Figure S2B). While FIT was significantly correlated with age at diagnosis (Pearson's R = −0.59, p = 0.042), we observed a stronger association with our PNS suggesting that in the specific context of individuals carrying *TP53* germline variants, the PNS may provide a more precise predictor of phenotypic variability (Fig. 2D and Figure S2B).

### Neoantigenic potential predicts attenuation of LFS severity

**Fig. 3:**
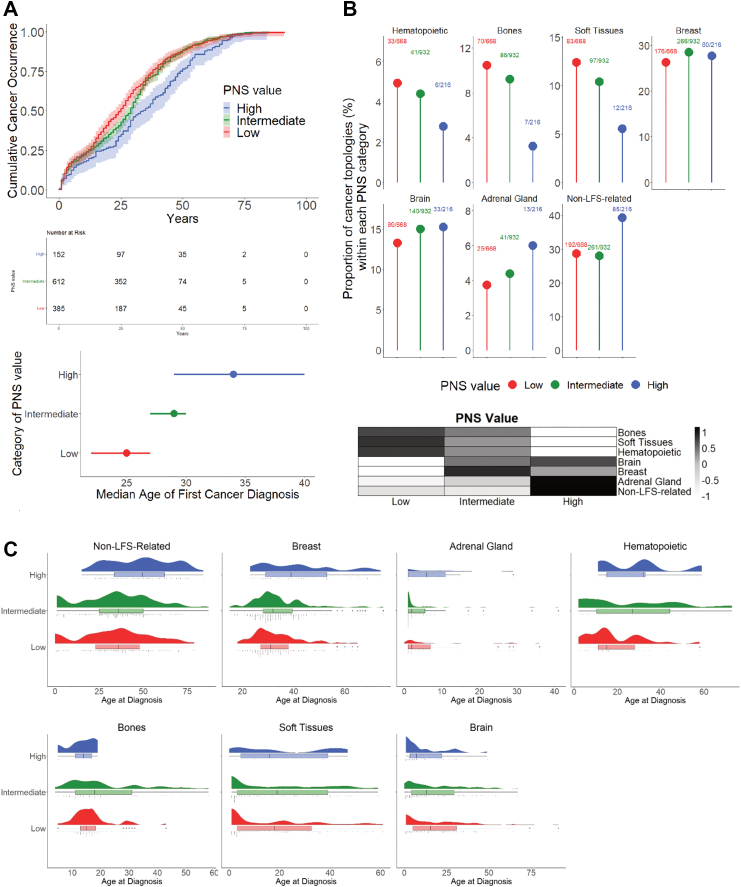
**Strength of PNS value is associated with an attenuated LFS phenotype. A.** Cumulative cancer occurrence patterns vary according to the PNS category. Individuals from NCI/IARC germline *TP53* database carrying a class A variant (n = 1426 with n = 1149 with age of first diagnosis) were separated into 3 groups based on the PNS value of the *TP53* variants, irrespective of the variant's hotspot status. PNS categories: High for PNS >2 (n = 152), Intermediate for 1≤ PNS ≤2 (n = 612), and Low for PNS <1 (n = 385). The inverted Kaplan–Meier curves represent the age of first cancer diagnosis for each individual with age information (top panel). A global Log-rank test was performed to assess differences between the curves (p = 0.0008). Multiple pairwise Log-rank tests were performed to compare PNS groups, with a Bonferroni correction applied: Low vs. High, Bonferroni-adjusted p = 0.0009; High vs. Intermediate, Bonferroni-adjusted p = 0.0019. Number at risk table is displayed below the graph. The bottom panel represents the median age of first cancer diagnosis (dots) along with confidence interval at 95% (bars asides the dots). **B.** Distribution of cancers' topology vary according to the PNS category. The typical LFS topologies are displayed (hematopoietic system, bones, soft tissues, breast, brain and adrenal gland) and non-LFS-spectrum topologies are grouped. For each cancer topology, the ratio of number of cases (n) over the total number of cancers analysed within each PNS value group (N) is indicated on the top of the dot representing the percentage of cancer topology. Total number of cancers analysed n = 1,816, with L PNS, n = 668; I PNS, n = 932; H PNS, n = 216. Multiple pairwise comparisons of proportion of topologies between the different PNS categories are performed using logistic model based on Chi-square statistic to extract risk ratio (RR) and a FDR-adjusted p-value (correction for multiple comparisons using Benjamini-Hochberg method). Significant risk ratio High vs. Low PNS: Bones [RR 0.29, FDR-adjusted p = 0.02], Soft Tissues [RR 0.41, FDR-adjusted p = 0.02], Non-LFS-spectrum [RR 1.61, FDR-adjusted p = 0.02]. Heatmap (bottom panel) summarises cancer topology distribution from top panel (normalisation in row by cancer topologies, and colour scale represents enrichment scores. **C.** Age distributions of cancers of different topologies vary according to the PNS category. The rain–cloud plots display (1) a density plot showing distribution of age of diagnosis of cancers, (2) a box plot showing median age of diagnosis as well as quartiles and outlier, and (3) a dot plot showing every cancer analysed. Multiple pairwise comparison of median age of cancer is performed between PNS groups for each cancer topology by running Wilcoxon rank sum tests with a Benjamini-Hochberg correction. Significant differences have been determined for breast cancer (High vs. Low, age 39 vs. 31, FDR-adjusted p = 0.0006; High vs. Intermediate, age 39 vs. 32, FDR-adjusted p = 0.0009) and non-LFS-spectrum cancers (High vs. Low, age 49.5 vs. 36, FDR-adjusted p < 0.0001; High vs. Intermediate, age 49.5 vs. 36, FDR-adjusted p < 0.0001).

When considering all individuals with class A variants (n = 1426) PNS predicted a significant 9 year delay in the median age at cancer diagnosis between the H PNS group (34 years [95% CI, 29–40]), vs. the L PNS group (25 years [95% CI, 22–27]); pairwise Log-rank test, Bonferroni-adjusted p = 0.0009), with the I PNS group showing an intermediate median age (29 years [95% CI, 27–30]; pairwise Log-rank test Bonferroni-adjusted p = 0.0019) (Fig. 3A).

Cancer-specific analyses revealed that the different components of the LFS tumour spectrum were not equally influenced by variant's predicted immunogenicity. When comparing individuals with H vs. L PNS variants, a significant decrease was observed in the proportions of osteosarcoma [RR 0.29, FDR-adjusted p = 0.02, pairwise Chi-square test] and soft tissues sarcoma [RR 0.41, FDR-adjusted p = 0.02, pairwise Chi-square test], as well as a tendency for hematopoietic malignancies. In contrast, the proportion of cancers not typically associated with the LFS spectrum were increased [RR 1.61, FDR-adjusted p = 0.02, pairwise Chi-square test]. PNS did not appear to significantly influence the proportion of breast, brain cancers or adrenal cortical carcinomas (Fig. 3B). The median age at diagnosis of most cancers also varied with PNS, with significant differences for breast cancer (H PNS vs. L PNS, 39 vs. 31 years, FDR-adjusted p = 0.0006; H PNS vs. I PNS, 39 vs. 32 years, FDR-adjusted p = 0.0009, Wilcoxon rank sum test) and non-LFS-spectrum cancers (H PNS vs. L PNS, 49.5 vs. 36 years FDR-adjusted p < 0.0001; H PNS vs. I PNS, 49.5 vs. 36 years, FDR-adjusted p < 0.0001, Wilcoxon rank sum test) (Fig. 3C). Also, variants with H PNS values were associated with a tendency for increased latency of adrenal cortical carcinomas and hematopoietic malignancies, whereas the age distribution of osteosarcoma, soft tissues sarcoma and brain tumours were similar across the 3 PNS groups (Fig. 3C). This selective attenuation of the LFS phenotype recapitulated the phenotypic characteristics of individuals carrying class B or C variants, who tended to present with less severe and more heterogeneous LFS phenotypes than those with class A variants.^14^ In particular, the cumulative cancer occurrence in individuals with H PNS class A variants was similar to that in individuals with class B variants (Figure S3A). Likewise, the shifts in cancer patterns in L vs. H PNS class A variants groups resembled those previously reported between class A vs. class B or C variant groups (Figure S3B and C). However, correlations between PNS and cancer phenotypes could not be evaluated in individuals carrying class B and C variants due to low numbers.

### Validation of association between neoantigenic potential and phenotype attenuation in LFS cohorts

These observations were repeated in the validation cohort (patients carrying a class A variant from French and German clinical LFS registries, n = 339).^1^^,^^13^ The validation cohort included 89 distinct class A variants, 63 of which overlapped with those identified in the NCI/IARC dataset, whereas 26 were unique to this cohort (Figure S4A). Despite shared hotspot variants, the relative frequencies of all variants differed between datasets, highlighting complementary variant distributions across the discovery and validation cohorts (Figure S4B). Significant differences were observed in cancer accrual between patients with H vs. L PNS class A variants (median age at first cancer diagnosis: 31 years [95% CI 24–38] in H PNS vs. 25 years [95% CI 21–28] in I PNS, and 23 years [95% CI 17–27] in L PNS, respectively; global Log–Rank test, p = 0.03) (Figure S4C). This cohort also recapitulated the tumour patterns observed in NCI/IARC *TP53* database, although small numbers precluded statistical significance (Figure S4D).

### Patient-level HLA-I profiles support association between neoantigenicity and phenotypic attenuation

**Fig. 4:**
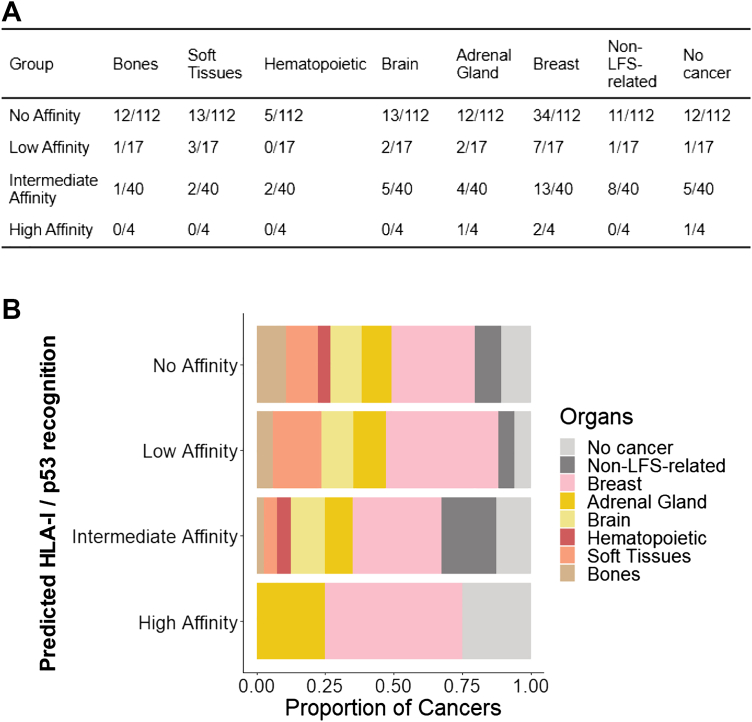
**Strong predicted neoantigenic response determined by individual HLA-I genotyping of LFS patients correlates with attenuated LFS phenotype. A.** Repartition of cancer topologies within each group of predicted neoantigenic response. The 6 HLA-I alleles types combined with the *TP53* variant identity were used to give an individual score for each patient, corresponding to the strongest affinity of any of his 6 HLA-I for the *TP53* variant carried. LFS patients (n = 173) were separated into 4 categories: high for affinity ≤10 nM (n = 4), intermediate for 10 nM > affinity ≤250 nM (n = 40), low for 250 nM > affinity <500 nM (n = 17), and no affinity when no HLA-I is predicted to interact with the variant (n = 112). First cancers are analysed (n = 169). **B.** The proportions of cancer topologies vary among groups of LFS patients separated based on the predicted neoantigenic response.

The PNS provides a population-level estimate, but individual variability depends on HLA-I genotype. Therefore, we next examined individual HLA-I genotypes in combination with *TP53* variants and clinical data. HLA-I genotyping data from 173 patients of the validation cohort^1^^,^^13^ showed that 65% (122) had no predicted HLA-I match at MAS ≤500 nM, whereas only 4 (2%) had hat least one high affinity HLA-I/variant peptide pair (MAS ≤10 nM) (Fig. 4A). Cancer spectrum differed according to MAS strata (High, Intermediate, Low or No Affinity), with members of Low and No Affinity groups recapitulating the broad LFS spectrum (cancers of the adrenal gland, brain, bones, soft tissues, breast and hematopoietic system) whereas Intermediate and High groups presented with attenuated LFS phenotypes characterised by a high proportion of cancer-free or non-LFS spectrum cancers and a low proportion of cancers of the bones and soft tissues (Fig. 4B). Thus, although high or intermediate affinity HLA-Is were rare in this LFS cohort, their presence was associated with tumour type-specific phenotypic attenuation which recapitulated the one observed in the NCI/IARC *TP53* database and the validation cohort.

## Discussion

Our results indicate associations between neoantigenic properties of functionally-impaired p53 protein variants and patterns of cancer risk in individuals with LFS. They suggest that pathogenic *TP53* germline variants may elicit a neoantigenic immune response, that potentially delays occurrence and selectively attenuates cancers of the LFS spectrum. Thus, neoantigenic differences could contribute to the broad clinical heterogeneity observed among individuals with LFS. Our results confirm and extend to germline variants the fitness selection model proposed by Hoyos et al.^9^ for somatic *TP53* variants, postulating that cancer-associated variants are selected to optimally solve an evolutionary trade-off between oncogenic potential and neoantigen immunogenicity. Hoyos et al. also observed that variants with lower combined fitness, integrating functional and immune components, was associated with later tumour onset in individuals with germline *TP53* variants, supporting the notion of an immune contribution to LFS variability. Consistently, our analysis shows that severely functionally disrupted (class A) variants with higher neoantigenic potential are associated to delayed tumour onset and a reduced incidence of classical LFS-associated cancers (Fig. 3). We show that the predicted neoantigenic score (PNS) was inversely correlated with immune fitness score from Hoyos et al.^9^ (Figure S2B), and that the most severely functionally disrupted *TP53* variants (class A) exhibited the highest PNS values (Fig. 1B). Similarly, Fortuno et al. recently reported lower immune fitness for pathogenic *TP53* variants.^16^ This inverse relationship supports the notion that pathogenic variants may elicit stronger immune recognition pressure than benign variants. Notably, the most commonly represented “hotspots” LFS variants tend to correspond to gaps in the HLA-I neoantigenic repertoire, which might allow these variants to escape immune recognition and cause severe LFS phenotypes. Thus, the *TP53* variant spectrum observed in individuals with LFS may reflects the combined effects of preferential mutagenesis, positive selection of severely impaired protein variants and negative selection of highly neoantigenic variants.

Whereas there is strong evidence that somatic *TP53* variants can elicit such a sustained and specific T-cell response, this has not yet been demonstrated in patients with germline *TP53* variants. A key question is to determine how neoantigenic variants escape central or peripheral tolerance during development. Our results are consistent with the notion that, under normal circumstances, p53 protein levels, either wild-type or mutant, are tightly regulated and kept at low levels during development and early life, only rising during stress or oncogenic transformation. Whereas LFS tumours often show stabilised and overexpressed mutant p53, whether this accumulation takes place in normal LFS cells and tissues is still a matter of debate.^17^^,^^18^ Loss of the wild-type allele (LOH) appears to be a critical event in LFS tumorigenesis. For instance, Light et al. demonstrated that early LOH with gain of the mutant *TP53* allele is a common feature across LFS tumours, highlighting its role as an early driver of tumour evolution.^19^ Complementing this, Agarwal et al. showed in LFS patient-derived fibroblasts, that mutant p53 accumulates only after LOH, providing a selective advantage to the affected cells.^20^ Thus, LOH may constitute the critical event causing mutant p53 to become exposed and available for neoantigenic presentation and the variant may therefore be seen by the immune system as “somatic”. Consistent with this interpretation, we noted that, among the predictors of neoantigenicity, Amplitude, a metrics that evaluates the difference in affinity between a neoantigen and the corresponding wild-type peptide,^15^ was only poorly correlated with age at cancer onset, in contrast with other predictors (Figure S2A). This finding suggests that the difference in HLA-I binding between wild-type and mutant peptides may not be a major determinant of its neoantigenicity, and that central tolerance to these peptides could be incomplete during development.

Our results suggest that immune attenuation does not equally affect all types of cancers within the LFS spectrum (Fig. 3B). Whereas high PNS variants appeared to be associated with delayed onset of most cancers, cancer risk was significantly reduced only for osteosarcoma and soft-tissue sarcoma. In contrast, the risk of adrenal cortical carcinoma and breast cancer appeared to remain unaffected. These differences might be caused by several mechanisms, including immune privilege and restricted access to specific types of cancers, default of certain cancers in processing the relevant neoantigenic peptides, or the need for tissue-specific additional signals to trigger full T-cell activation. Mutant p53 has been associated with profound alterations of immune regulations with documented effects on macrophages, dendritic cells, natural killer cells, T cells, and B cells.^21^ Such effects may enable certain cancers to escape immune surveillance despite the presence of a potent neoantigen. An alternative explanation is that neoantigenic attenuation might be inversely proportional to the number of LFS cancer initiating events occurring in different tissues. Indeed, the phenotypic attenuation caused by variant neoantigenicity is remarkably similar to the one caused by variant functionality. In other words, tumour phenotypes in individuals with class A (most severe) variants with high or intermediate PNS are very similar to those with class B or C (less severe) variants with low PNS (Figure S3). We hypothesise that class B or C variants may support the emergence of less tissue-specific LFS cancer-initiating cells than class A variants. Similar to functional attenuation, neoantigenic attenuation may operate by controlling the pools of such initiating cells, causing a significant risk reduction in tissues where these pools are rate-limiting, but not in tissues in which a high number of initiating events enables the persistence of initiating cells even in the face of immune attenuation. As a result, the cancers that appear less sensitive to neoantigenic attenuation are precisely those that are the most frequent in children and adult LFS patients, namely, adrenal cortical and breast cancers, respectively.

These results may have implications for predicting cancer risk and surveillance in individuals carrying germline *TP53* mutation. Although most patients with severe LFS may lack high-affinity HLA-I allele to their variants, HLA-I typing could provide an explanatory variable in subjects who present attenuated, atypical or even no LFS phenotype despite the presence of a pathogenic variant. In this respect, neoantigenicity may contribute to inter-individual and intra-familial heterogeneity in LFS presentation. In the long term, and after appropriate clinical evaluation, HLA-I typing might help refine personalised risk assessment and surveillance strategies. While variant neoantigenicity could potentially inform individualised immuno-preventive and immunotherapeutic approaches, these applications remain hypothetical and require validation. Importantly, neoantigenicity alone is unlikely to account for the full spectrum of phenotypic variability observed in LFS. Other factors, such as *TP53* haplotypes, genetic modifiers, environmental exposures, and lifestyle influences, are likely to contribute to both inter- and intra-familial heterogeneity. Integrating these dimensions with immunogenetic profiling may further improve genotype–phenotype correlations in the future.

The limitations of this work are, first and foremost, its observational and correlative nature. Whereas it reveals a strong correlation between LFS latency, risk and variant neoantigenicity, it does not demonstrate an effective cancer protective or suppressive anti-mutant p53 T-cell response in patients with germline *TP53* variants. Further experimental and clinical studies are required to evaluate the precise extend, dynamics and suppressive efficacy of such an immune response. Another limitation is that it is based on predicted interactions between HLA-I and every possible 9-mer peptide derived from missense *TP53* variants, without taking into account which peptides may actually be effectively cleaved and processed by the immunoproteasome and by cell-specific intermediate proteasomes. In our analysis, the usage of current standard peptide cleavage prediction models proved to restrict the set of peptides deemed as potential neoantigens and did not increase the accuracy of phenotypic correlations, suggesting that these models may not adequately capture the diversity of peptide proteolytic mechanisms that lead to the recognition of p53 protein variants. Another limitation is that our approach is currently restricted to missense variants. While certain splice, nonsense, or frameshift/indel variants could potentially generate non-wild-type peptides, additional modelling and experimental validation would be required to accurately assess their neoantigenic potential. Another limitation concerns cohort representativeness. Both the NCI/IARC and LFS registries are enriched for individuals of European and American ancestry, with limited representation of Asian and African populations. As the PNS relies on global rather than individual HLA-I frequencies, our predictions may not fully capture cohort-specific HLA-I diversity. Expanding individual-level HLA-I genotyping will help refine and generalise our predictions.

Predicted neoantigenic qualities of pathogenic *TP53* variants are correlated with variant frequency and cancer latency in individuals with LFS. This observation supports the notion that variants can elicit a T-cell immune response leading to an attenuation of the cancer risk in LFS patients. Variant neoantigenic prediction scores may help in assessing cancer risk in individuals with pathogenic *TP53* variants and could contribute to understanding inter-individual variability in the LFS spectrum.

## Contributors

Conceptualization: EM, OM, DH, BDG, AJL, CPK, GB, and PH, Methodology: EM, OM, DH and BDG, Software: EM and OM, Resources: OM, CPK, and GB, Data curation: CF, SB, MV, CMD, SC, JB, BF, RO, and JFD, Formal analysis: EM and OM, Investigation: EM, OM, JG, CF, and PH, Visualization: EM and JG, Writing – original draft: EM and PH, Writing – review and editing: EM, AJL, CPK, GB and PH. Funding acquisition: EM, TF, CPK, GB and PH, Project administration: EM, CPK, GB, and PH, Supervision: CPK, GB, and PH.

EM, OM and PH have directly accessed and verified the underlying data reported in the manuscript. EM and PH were responsible for the decision to submit the manuscript. All authors read and approved the final version of the manuscript.

## Data sharing statement

Aggregated genotype–phenotype data extracted from the public *TP53* database are available at https://tp53.isb-cgc.org/. Clinical registry data used in this study were obtained from previously published cohorts (Bougeard et al., J Clin Oncol 2015; Penkert et al., J Hematol Oncol 2022). Anonymised genomic and clinical data from these registries, including HLA-I typing and *TP53* variant information, are summarised in Table S3. Neoantigenic scores, developed using NetMHCpan 4.1 and the Allele Frequency Net Database, are available in Table S1; the list of 145 HLA-I alleles studied is provided in Table S2. The code used for neoantigenic score prediction, data analysis and statistics is available from the corresponding author upon reasonable request for academic research purposes.

## Declaration of interests

The authors declare that they have no conflicts of interest.
