# Supplementary material for: Neoantigenic properties of TP53 variants influence cancer risk in individuals with Li-Fraumeni syndrome
Source: eBioMedicine. 2025 Dec 9;123:106065. doi: 10.1016/j.ebiom.2025.106065 (PMC12752760; doi:10.1016/j.ebiom.2025.106065)
Supplement: Supplementary Figures [file mmc1.pdf]

Figure S1

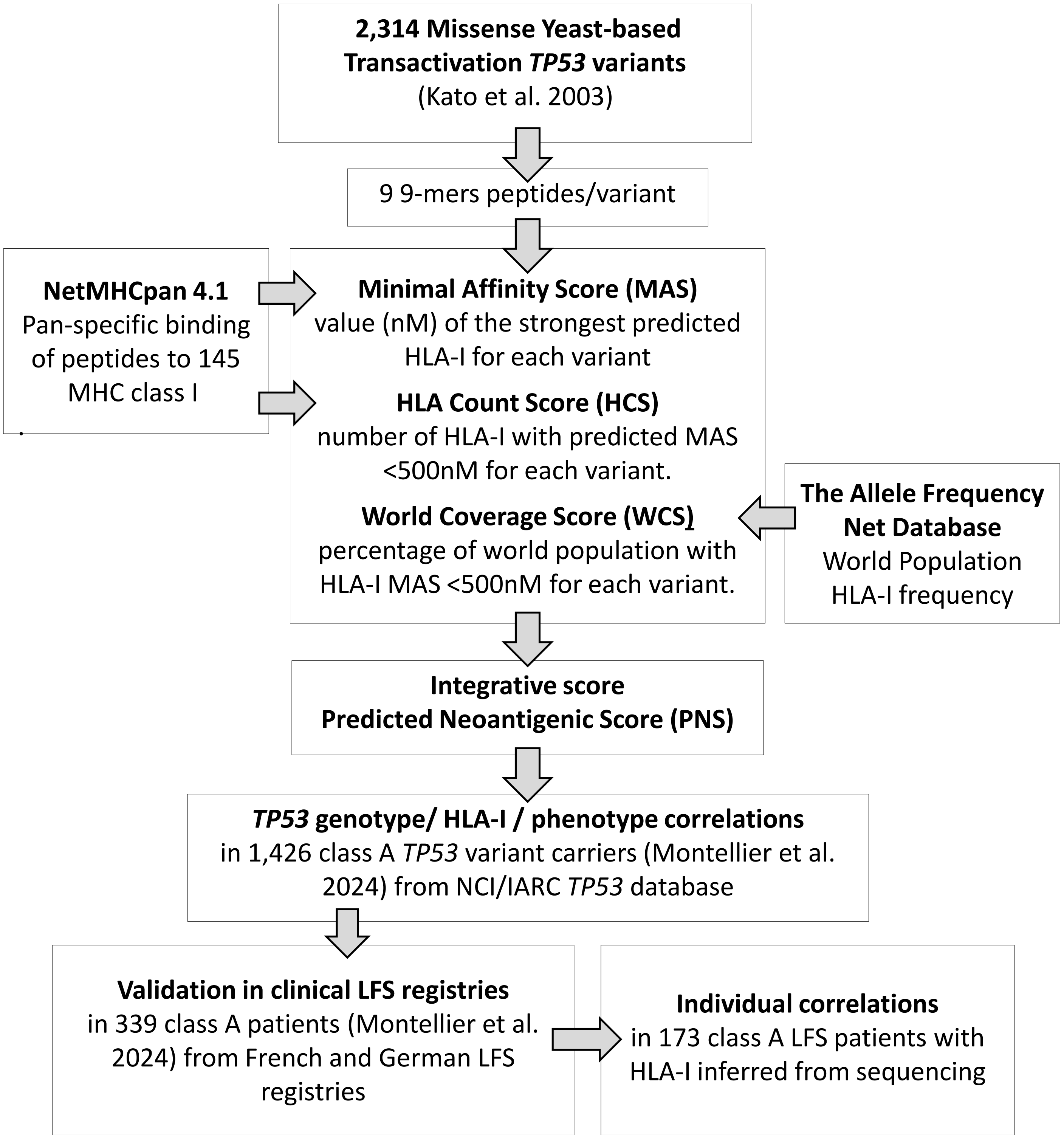

2,314 Missense Yeast-based Transactivation TP53 variants
(Kato et al. 2003)
9 9-mers peptides/variant
NetMHCpan 4.1
Pan-specific binding of peptides to 145 MHC class I
Minimal Affinity Score (MAS)
value (nM) of the strongest predicted HLA-I for each variant
HLA Count Score (HCS)
number of HLA-I with predicted MAS <500nM for each variant.
World Coverage Score (WCS)
percentage of world population with HLA-I MAS <500nM for each variant.
The Allele Frequency Net Database
World Population HLA-I frequency
Integrative score
Predicted Neoantigenic Score (PNS)
TP53 genotype/ HLA-I / phenotype correlations
in 1,426 class A TP53 variant carriers (Montellier et al. 2024) from NCI/IARC TP53 database
Validation in clinical LFS registries
in 339 class A patients (Montellier et al. 2024) from French and German LFS registries
Individual correlations
in 173 class A LFS patients with HLA-I inferred from sequencing

Figure S2

A

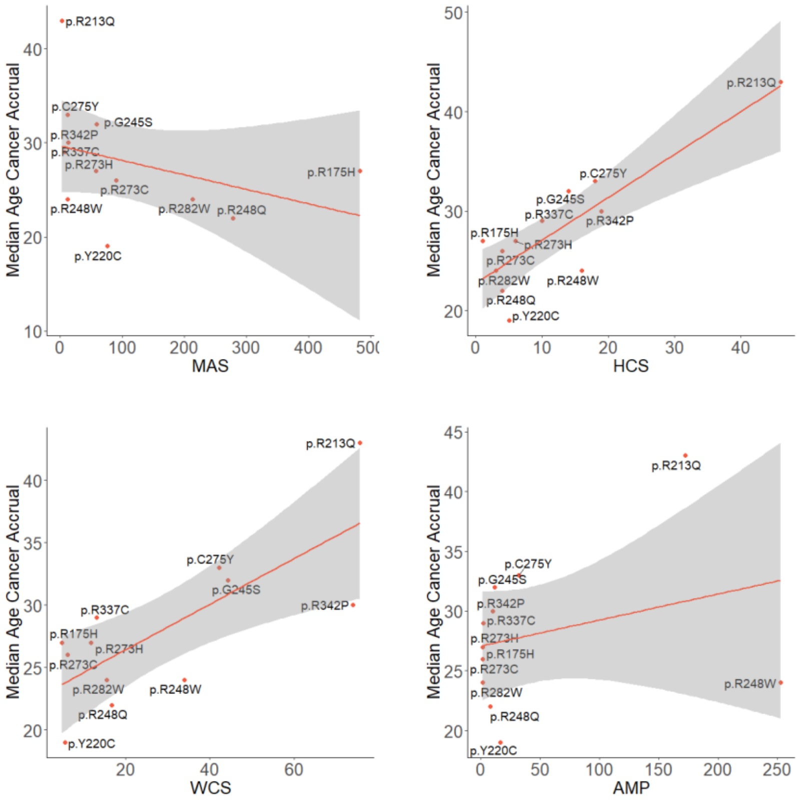
Median Age Cancer Accrual
MAS
HCS
WCS
AMP
p.R213Q
p.C275Y
p.G245S
p.R342P
p.R337C
p.R273H
p.R175H
p.R273C
p.R248W
p.R282W
p.R248Q
p.Y220C

B

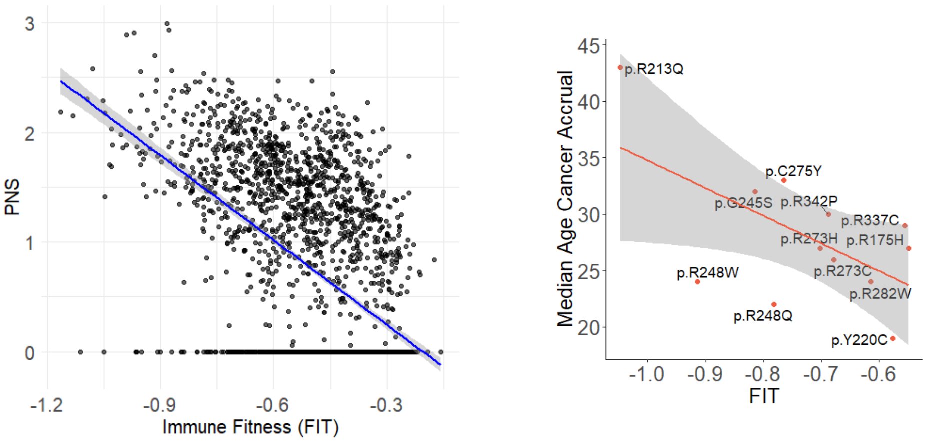
PNS
Immune Fitness (FIT)
Median Age Cancer Accrual
FIT
p.R213Q
p.C275Y
p.G245S
p.R342P
p.R337C
p.R273H
p.R175H
p.R248W
p.R273C
p.R282W
p.R248Q
p.Y220C

Figure S3

A

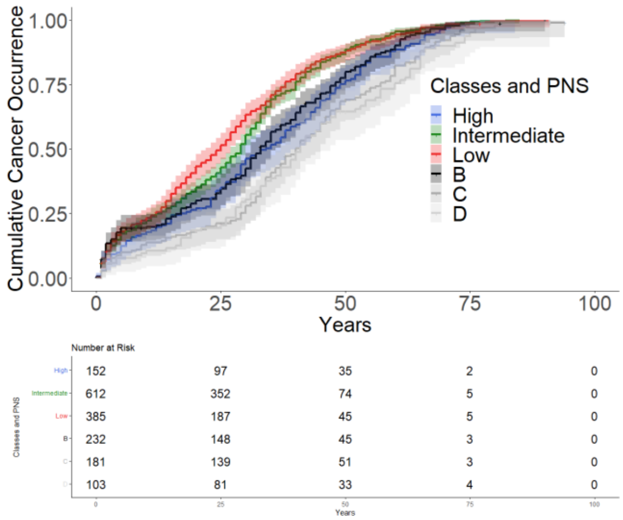

Comparison	Adjusted_P_Value	Significance
High vs. Intermediate	7.63e-03	**
Intermediate vs. Low	1.00e+00	NS
Low vs. High	3.54e-03	**
High vs. B	1.00e+00	NS
High vs. C	1.13e-01	NS
High vs. D	5.82e-02	NS
Intermediate vs. B	8.06e-03	**
Intermediate vs. C	4.07e-11	***
Intermediate vs. D	1.98e-08	***
Low vs. B	4.56e-03	**
Low vs. C	9.85e-11	***
Low vs. D	4.76e-08	***

B

C

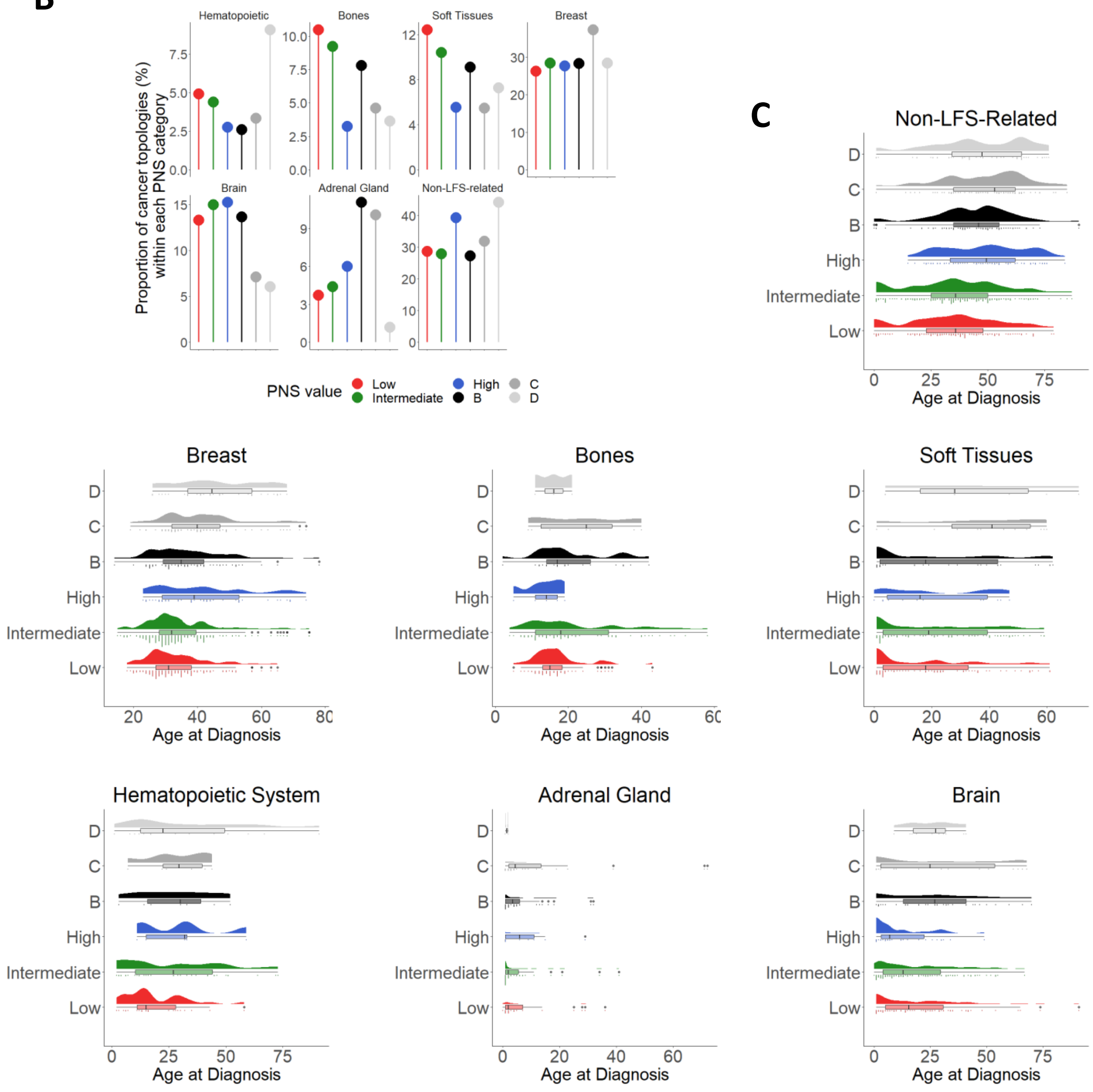

Figure S4

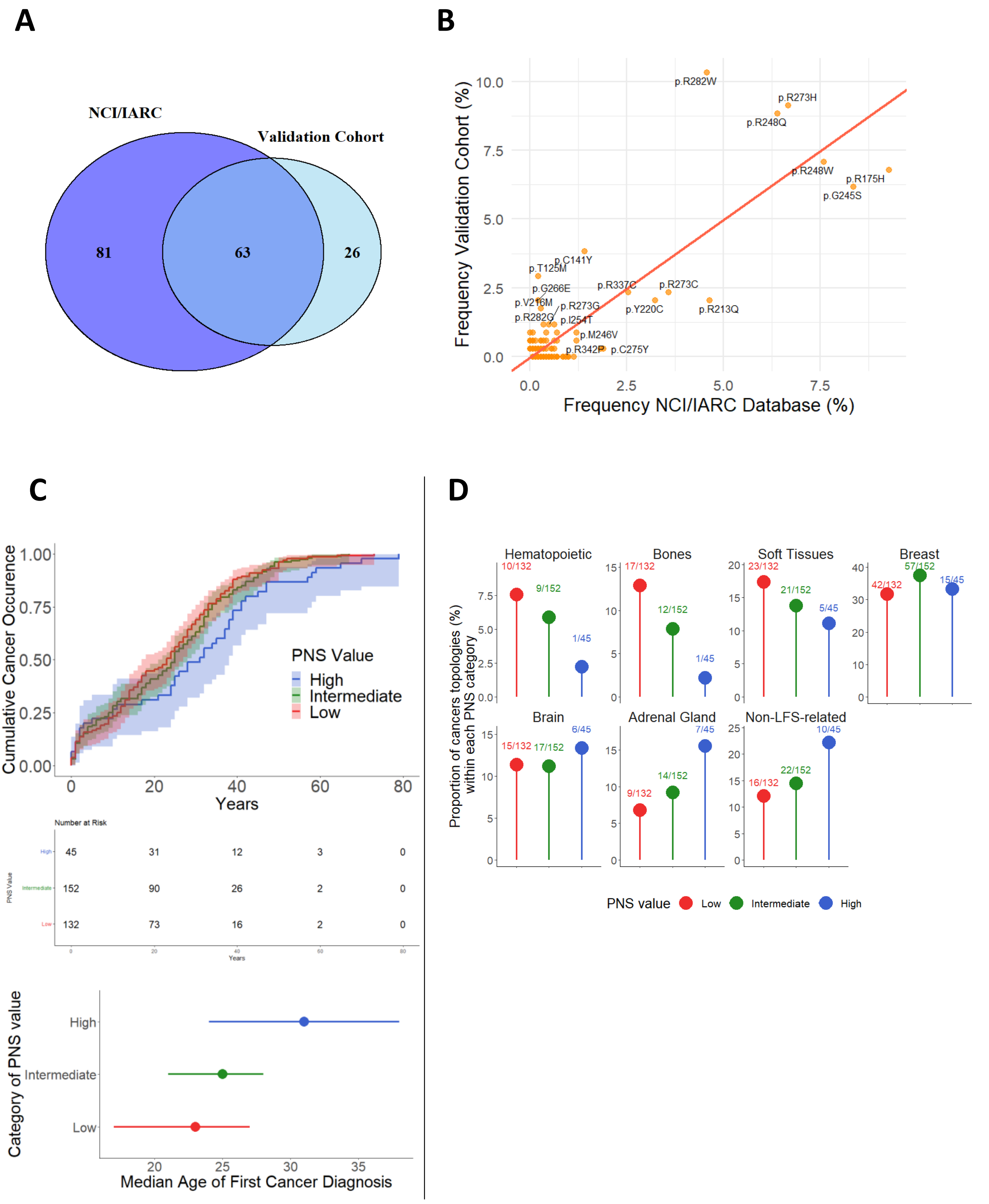
A
NCI/IARC
Validation Cohort
81
63
26
B
Frequency Validation Cohort (%)
Frequency NCI/IARC Database (%)
p.R282W
p.R273H
p.R248Q
p.R248W
p.R175H
p.G245S
p.C141Y
p.T125M
p.G266E
p.R337C
p.R273C
p.V216M
p.R273G
p.Y220C
p.R213Q
p.R282G
p.I254T
p.M246V
p.R342P
p.C275Y
C
Cumulative Cancer Occurence
Years
PNS Value
High
Intermediate
Low
Number at Risk
PNS Value
High 45 31 12 3 0
Intermediate 152 90 26 2 0
Low 132 73 16 2 0
Years
Category of PNS value
High
Intermediate
Low
Median Age of First Cancer Diagnosis
D
Proportion of cancers topologies (%) within each PNS category
Hematopoietic
10/132
9/152
1/45
Bones
17/132
12/152
1/45
Soft Tissues
23/132
21/152
5/45
Breast
42/132
57/152
15/45
Brain
15/132
17/152
6/45
Adrenal Gland
9/132
14/152
7/45
Non-LFS-related
16/132
22/152
10/45
PNS value
Low
Intermediate
High

SUPPLEMENTARY FIGURES LEGEND

Figure S1: Flowchart of the study: generation of the Predicted Neoantigenic Score (PNS) for *TP53* variants and subsequent genotype-phenotype correlations in individuals carrying germline *TP53* variants.

Figure S2: Correlation of neoantigenic scores with median age of first cancer diagnosis for the 12 *TP53* class A more frequent variants (supplemental information related to Figure 2D).
A. Correlation of median age of first cancer diagnoses and the 3 scores used to build the PNS (MAS, HCS and WCS), and the amplitude score (AMS). For each *TP53* variant, the median age of first cancer was plotted against the different scores. The linear regression line is displayed in orange, and a Spearman correlation test was performed to assess relationships (MAS: rho = -0.63, p=0.0276; HCS: rho = 0.67, p=0.0168; WCS: rho = 0.61, p=0.03367; AMS: rho = 0.25 p=0,4349). **B.** Relationship between the PNS and the immune fitness score (FIT) developed by Hoyos et al. (Nature, 2022) (Spearman's rho=-0.50, p < 0.0001), left panel. The right panel represents the correlation of median age of first cancer diagnoses and the FIT. The linear regression line is displayed in orange, and a Pearson correlation test was performed to assess relationship (R = -0.59, p=0.0420).

Figure S3: Class A LFS individuals carrying a *TP53* variant with high PNS have shared traits with individuals carrying class B or C variant (supplementary information related to Figure 3).
A. Cumulative cancer occurrence of class B, C and D individuals compared with class A separated into high, intermediate and low PNS. Individuals from NCI/IARC germline *TP53* database carrying a single missense variant (n=2,129, with n=1,665 individuals with age of first diagnosis) were separated into class A (n=1,149), B (n=232), C (n=181) and D (n=103). Class A were sub-classified into 3 groups based on the PNS. PNS categories: High for PNS >2 (n=152), Intermediate for 1-2 (n=612), and Low for PNS <1 (n=385). The inverted Kaplan-Meier curves represent the age of first cancer diagnosis for each individual with age information (top left panel). Number at risk table is displayed below the graph. A Log-rank test was performed to assess differences between the curves (p<0.0001). Pairwise Log-rank tests were performed to assess comparisons between classes and PNS groups with a Bonferroni correction (Adjusted-p-value) (right panel). **B.** Distribution of cancers' topology vary according to the classes and PNS categories. The typical LFS topologies are displayed (hematopoietic system, bones, soft tissues, breast, brain and adrenal gland) and other non-LFS-related topologies are grouped. Total number of cancer analysed n=2,526 (A H PNS, n=216; A I PNS, n=932; A L PNS, n=668; B, n=307; C, n=238; D, n=165). **C.** Age distributions of cancers of different topologies vary according to the classes and PNS categories. The rain-cloud plots display (1) a density plot showing distribution of age of diagnosis of cancers, (2) a box plot showing median age of diagnosis as well as quartiles and outlier, and (3) a dot plot showing every cancer analysed.

Figure S4: Strength of PNS value is associated with an attenuated LFS phenotype in a validation cohort of LFS patients.
A. Overlap between class A *TP53* variants identified in the NCI/IARC germline dataset (n = 144) and those observed in the validation cohort from the French and German LFS registries (n = 89). **B.** Distribution of relative frequencies within class A *TP53* variants across the two datasets (NCI/IARC germline dataset and validation cohort). **C.** Cumulative cancer occurrence patterns vary according to the PNS category. Individuals from the validation cohort (LFS patients), carrying a class A variant (n = 339, with n=329 with a cancer diagnosis) were separated into 3 groups based on the PNS value of the *TP53* variants. PNS categories: High for PNS >2 (n=45), Intermediate for 1≤ PNS ≤2 (n=152), and Low for PNS <1 (n=132). The inverted Kaplan-Meier curves represent the age of diagnosis of the first cancer for each individual (top panel). A Log-rank test was performed to assess differences between the curves (p = 0.03). Number at risk table is displayed below the graph. The bottom panel represents the median age of first cancer diagnosis (dots) along with confidence interval at 95% (bars asides the dots). **D.** Distribution of cancers' topologies vary according to the PNS category. The typical LFS topologies are displayed (hematopoietic system, bones, soft tissues, breast, brain and adrenal gland) and other non-LFS-related topologies are grouped. First cancer of patients are analysed (n=329, with L PNS, n=132; I PNS, n=152; H PNS, n=45). For each cancer topology, the ratio of number of cases (n) over the total number of cancers analysed within each PNS value group (N) is indicated on the top of the dot representing the percentage of cancer topology.

SUPPLEMENTARY TABLES LEGEND

Table S1: 2,314 *TP53* missense variants along with neoantigenic scores.
Legend of the columns: ProtDescription: TP53 variant; YTA_class: Functional classes (A, B, C, D) from Montellier et al. 2024; Codon_number: p53 codon; MAS: Minimal Affinity Score (nM); HCS: HLA-I Count Score (HCS); WCS: World Coverage Score (%); AMS: Amplitude Score; Normalised MAS: MAS values scored from 0 to 1; Normalised HCS: HCS values scored from 0 to 1; Normalised WCS: WCS values scored from 0 to 1; PNS: Predicted Neoantigenic Score, corresponding to the sum of Normalised MAS, Normalised HCS, and Normalised WCS; Category: PNS category (low, intermediate, high); HLA-I_[number]: Identity of HLA-I predicted to interact with a given TP53 variant.

Table S2: List of the 145 HLA-I studied and the number of the predicted *TP53* variants recognized.

Table S3: Dataset of LFS patients with *TP53* variant identity, HLA-I typing, and clinical data.
Legend of the columns: Unlabbeled_Patients: numbered from 1 to 173; A1, A2, B1, B2, C1, and C2: HLA-I typing of the patient; ProtDescription: TP53 variant carried by the patient; YTA_class: Functional classes (A, B, C, D) from Montellier et al. 2024; Age_Cancer_1: Age of the first cancer of the patient, or age of last follow-up if no cancer; Topology_Cancer_1: Tumor distribution of the first cancer of the patient.
